# Phacoemulsification practices: A comprehensive analysis of the surgical landscape in Sweden 2021–2022

**DOI:** 10.1111/aos.16754

**Published:** 2024-08-20

**Authors:** Johan Ursberg, Madeleine Zetterberg, Andreas Viberg

**Affiliations:** ^1^ Department of Clinical Neuroscience, Institute of Neuroscience and Physiology, Sahlgrenska Academy University of Gothenburg Gothenburg Sweden; ^2^ Division of Ophthalmology Aleris Healthcare Sweden Stockholm Sweden; ^3^ Department of Ophthalmology Sahlgrenska University Hospital Mölndal Sweden; ^4^ Department of Clinical Sciences/Ophthalmology Umeå University Umeå Sweden

**Keywords:** cataract surgery, cross‐sectional survey, phacoemulsification techniques, tilt and tumble

## Abstract

**Purpose:**

This cross‐sectional survey study aimed to explore the phacoemulsification techniques among Swedish cataract surgeons, and investigate the association between technique preferences and surgical outcomes, particularly posterior capsular rupture (PCR).

**Methods:**

A survey questionnaire was responded by 170 cataract surgeons and data from 192 494 cases, linked to the surgeons, were analysed from the Swedish National Cataract Registry (SNCR) for 2021–2022. Surgeons' demographic characteristics, surgical techniques and complications were assessed. Associations between surgical technique preferences and outcomes were analysed with binary logistic regression.

**Results:**

The chopping technique (stop and chop or direct chop) was favoured by 64.6% of surgeons, followed by divide and conquer (32.4%), and tilt and tumble (7.6%). Surgeons' annual caseloads varied widely (range 11–2687). No significant correlation was found between technique preference and PCR rates, which was consistently 0.5%–0.6% in all groups, except for a trend suggesting reduced risk with tilt and tumble. Mentoring activity (35.0%) and public surgical setting (40.3%) was highest in the direct chop group. Notably, 75% of the surgeries were performed by surgeons with more than 10 years' experience. Confounding factors, such as high‐volume surgeons having a low frequency of complications, have been accounted for in a logistic regression.

**Conclusion:**

This study provides insights into cataract surgery practices in Sweden and suggests that surgeons can choose their preferred approach without significantly affecting complication rates. This research also underscores the need for continued exploration of surgical practices and their impact on patient outcomes, particularly in the case of the tilt and tumble technique, which is less commonly employed.

## 1 INTRODUCTION

The introduction of phacoemulsification transformed cataract surgery from a daunting procedure with prolonged visual impairment into a patient‐friendly intervention, offering almost immediate visual recovery (Kelman, 1967). Since then, the landscape of phacoemulsification has evolved significantly, with a multitude of techniques emerging (Kang et al., 2021), each with its opponents and proponents (Park et al., 2013). These techniques range from classic techniques like divide and conquer, stop and chop, direct chopping and tilt and tumble to innovative approaches like femtosecond laser assisted surgery. The choice of technique often reflects the surgeon's preference, experience and training background. This prompts the question: which of these techniques are the most prevalent, and is there a superior approach among them?

This article highlights the various phacoemulsification techniques in cataract surgery and the importance of understanding these techniques for better outcomes and advancements in the field. A cross‐sectional survey was conducted among cataract surgeons in Sweden to identify commonly used techniques, variations among surgeons, their impact on outcomes and complications like PCR. The study compared the surgeons' reported practices with actual patient‐based data from the Swedish National Cataract Registry (SNCR) from 2021 to 2022. This allowed for comparison of self‐reported practices of cataract surgeons with objective patient‐based data.

## 2 MATERIALS AND METHODS

A cross‐sectional survey study was conducted to explore surgical techniques and trends among Swedish cataract surgeons. An electronic survey questionnaire was developed to gather information regarding surgical practices, techniques and trends. Invitations to participate in the survey were sent to all active cataract surgeons identified from the SNCR database (334 surgeons). Participation in the survey was voluntary, and informed consent was obtained from all participants. The survey was distributed in December 2022 and respondents were given 3 months to complete the survey. Follow‐up reminders were sent to enhance the response rate and 170 surgeons (50.9%) chose to participate. The study also aimed at comparing the self‐reported practices of cataract surgeons with patient‐based data from the SNCR, which maintains comprehensive records of cataract surgeries performed across the country. The survey encompassed inquiries pertaining to participants' surgical experiences; surgical educational guidance; favoured surgical techniques; and the clinical environment in which they operated, distinguishing between private and public settings.

Patient‐based data from the SNCR, which exclusively covers patients aged 8 years and above, containing detailed records of cataract surgeries performed nationwide, were obtained from 1 January 2021 to 31 December 2022. The SNCR data encompassed patient demographics, preoperative status, intraoperative difficulties and complications. A total of 285 294 cataract procedures were obtained from the SNCR; 192 494 cases (67.5%) were linked to the survey‐responding surgeons and could be included in the study. Exclusion criteria for procedures performed by surgeons enrolled in the study included: patients below 8 years of age, procedures by surgeons conducting fewer than 10 cases per year (*n* = 27), procedures by a single surgeon stating ‘other’ as preferred surgical technique (*n* = 1), procedures on patients with unspecified gender (*n* = 14), and procedures with unknown surgical ID (*n* = 2).

The survey results of each responding surgeon were matched with the surgical ID in SNCR so that the answers in the survey from a specific surgeon could be matched with the data in the register for all the cases this surgeon had performed during 2021–2022.

### 2.1 Surgical techniques

In the survey, each participating surgeon was requested to evaluate their preferred surgical technique from the given options and quantify the degree of utilization for the specified technique, see Figure S1. According to both an American (Bethke, 2022) and a European (ESCRS, 2021) cataract surgery technique survey, the following four techniques were known to be the most established techniques used to break the nucleus during phacoemulsification. Of the 170 responding surgeons in our survey, 169 stated one of the following techniques as their most favoured technique (Table 1).

1. The *divide and conquer* technique, recognized as the most classical approach, commences with the creation of a longitudinal groove in the cataractous lens. Subsequently, a second groove is sculpted perpendicular to the initial one. The phaco probe, together with the auxiliary instrument, is then used to separate the nucleus into four quadrants by pushing the instruments apart in the grooves, each subsequently phacoemulsified individually (Gimbel, 1991).
2. *Stop and chop* is a hybrid technique between *divide and conquer* and *horizontal chopping*. The procedure begins with nuclear cracking and then continues as a chop technique. It commences with the creation of a central groove. Just like in *divide and conquer*, the nucleus is then divided into two hemisections by cracking. This is followed by insertion of a chopper which is buried in the nuclear periphery and pulled towards the centre, resulting in the nucleus being chopped into bite‐size pieces which then can be phacoemulsified easily (Koch & Katzen, 1994).
3. *Direct chopping* techniques utilize manual instrument forces to segment the nucleus instead of making an initial groove. Using the phaco tip, the nucleus is grasped, and a chopping motion is applied to generate two planes of cracking, followed by phacoemulsification. Vertical and horizontal chops, representative of this approach, emphasize an energy effective technique for nucleus fragmentation and is also believed to cause less stress on the zonules than grooving techniques (Buratto, 1998; Chang, 2003; Jacob, 2019; Lee & Chang, 2023).
4. The *tilt and tumble* technique is a modified form of supracapsular phacoemulsification. It uses hydrodissection, where a cannula elevates the capsular rim, to release and tilt the nucleus partly out of the bag. Gentle irrigation generates a fluid wave beneath the capsular space, leading to nuclear pole tilting. Subsequent engagement and emulsification of the prolapsed pole are performed using the phaco probe. The remaining nucleus is then tumbled out and emulsified in a similar manner (Davis & Lindstrom, 2002; Lindstrom, 1993).

**TABLE 1:** Responses to questionnaire on cataract technique among Swedish surgeons.

Variable	Number (%)
Cataract surgeons, *n* = 165^a^	
Men	114 (69.1)
Women	51 (30.9)
Surgical setting, *n* = 170	
Public	102 (60.0)
Private	61 (35.9)
Both public and private	7 (4.1)
Surgical experience, *n* = 170	
0–1 year	8 (4.7)
2–5 years	26 (15.3)
6–10 years	34 (20.0)
>10 years	102 (60.0)
Surgical tutoring 2021–2022, *n* = 170	
Yes	42 (24.7)
No	128 (75.3)
Surgical technique, most favoured, *n* = 170/195^b^	
Divide and conquer	55 (32.4)
Stop and chop	76 (44.7)
Direct chop	50 (29.4)
Tilt and tumble	13 (7.6)
Other	1 (0.6)
Surgical technique, uses sometimes (regardless of how often), *n* = 170	
Divide and conquer	114 (67.1)
Stop and chop	127 (74.7)
Direct chop	74 (43.5)
Tilt and tumble	56 (32.9)
Other	16 (9.4)

^a^ Five surgeons with unknown gender.

^b^ Some surgeons had more than one favourite method, hence more than 170 answers to ‘most favoured technique’.

### 2.2 Statistical procedures

The survey allowed multiple preferred techniques and the statistical comparison applies to one preferred technique compared to the other technique. For categorical variables, the Chi‐squared test or Fisher's exact test were employed to assess associations and differences in proportions (%) between survey responses and SNCR data. Continuous variables were described by mean and standard deviation (SD) as well as median and range and statistical significance tested using non‐parametric tests (Mann–Whitney *U*‐test) in case of lack of normal distribution. A binary logistic regression was performed with posterior capsular rupture as dependent variable and the following variables as covariates: age, gender, preferred technique, surgical volume, experience, surgical setting, preoperative visual acuity, capsular staining and the use of capsular hooks. Statistical analysis was performed using spss version 28.0.1.1 (IBM, USA) and a *p*‐value less than 0.05 was considered significant.

## 3 RESULTS

**FIGURE 1:**
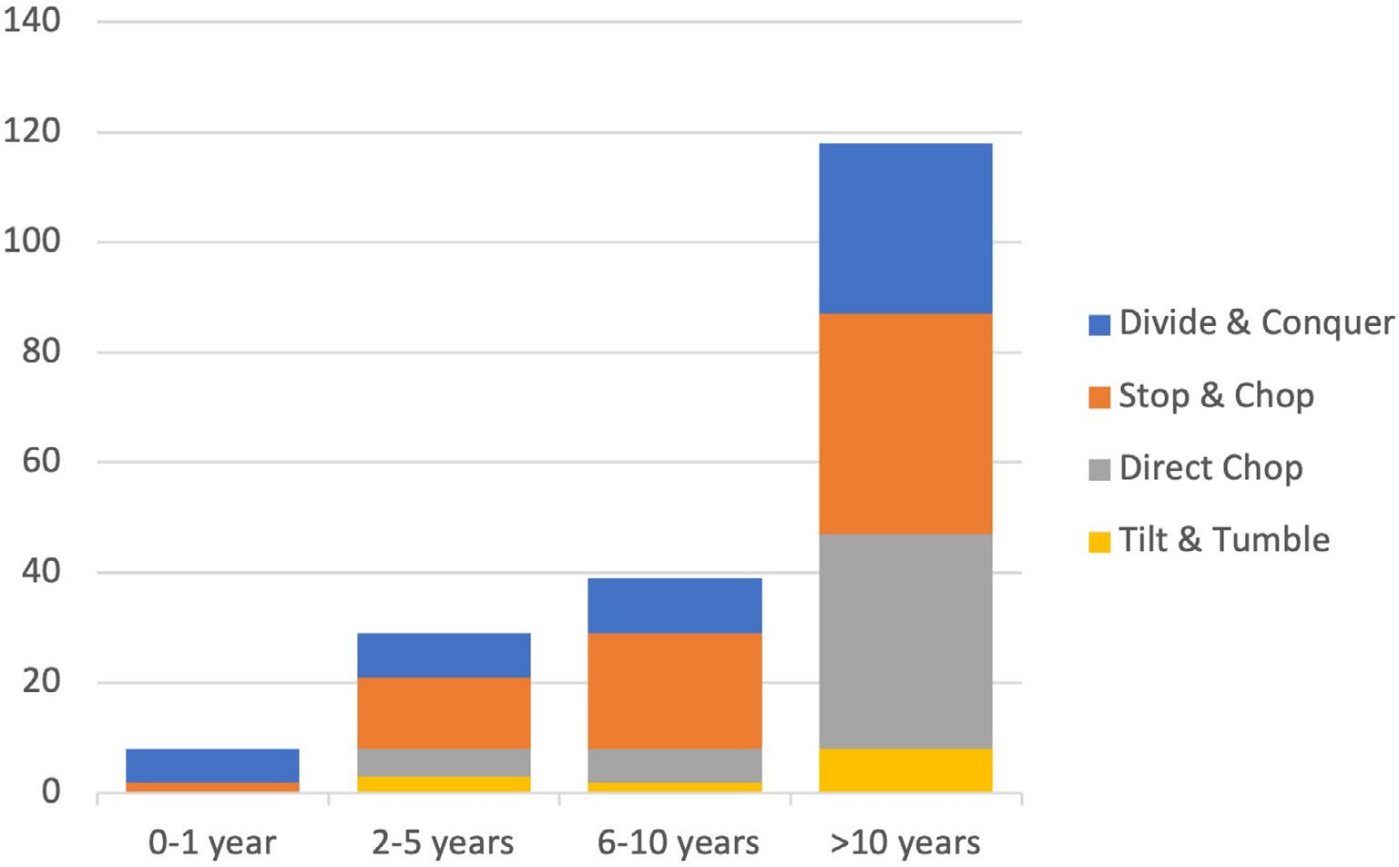
Preferred Surgical Techniques in Relation to Experience: This graphic depicts the distribution of favoured surgical techniques in relation to a surgeon's experience. The height of each bar correlates with the number of surgeons in each experience group and the colours represent the proportion preferring a particular technique. It was possible to have more than one favourite technique.

The survey encompassed 170 participating surgeons. Their demographic characteristics and surgical preferences are presented in Table 1. Among the respondents, 114 (69%) were males, 51 (31%) were females and five (3%) did not specify their gender. A significant portion of these surgeons, precisely 102 (60%), practised in public healthcare settings, and an equal number, 102 (60%), had accumulated more than a decade of experience. Surgeons in private practice had over 10 years of experience in 84% of the cases compared to 58% of the cases performed in public practice. Furthermore, 42 (24.7%) of the surgeons were actively engaged in surgical teaching (as teachers) during the 2021–2022 period. Some of the 170 surgeons (*n* = 20) declared multiple techniques as their favourite, resulting in 195 stated favoured techniques. We found that chopping techniques, including both stop and chop and direct chop approaches, were the dominant techniques used. The divide and conquer method was the next most commonly chosen, with 55 (32.4%) surgeons expressing a preference for it. In contrast, the tilt and tumble method (supracapsular phaco) was comparatively infrequently used, accounting for 13 (7.6%) of the favoured documented surgical approaches. The preferred surgical techniques in relation to experience is shown in Figure 1. Surgeons with less than 1 year of practice prefers divide and conquer and stop and chop exclusively. With increased experience, more techniques are introduced, but divide and conquer and stop and chop are still common techniques.

The survey‐responding surgeons performed 192 494 (67.5%) of the procedures during 2021 and 2022 and the non‐responding surgeons performed 92 539 (32.5%) during the same period. Data from the SNCR revealed some distinctions between these two groups; notably, non‐respondent surgeons had lower surgical volumes, practised more in a public setting, were more likely to perform other types of cataract surgery than ordinary phacoemulsification, including combined procedures, and had a slightly higher incidence of posterior capsular rupture, see Table S1.

**FIGURE 2:**
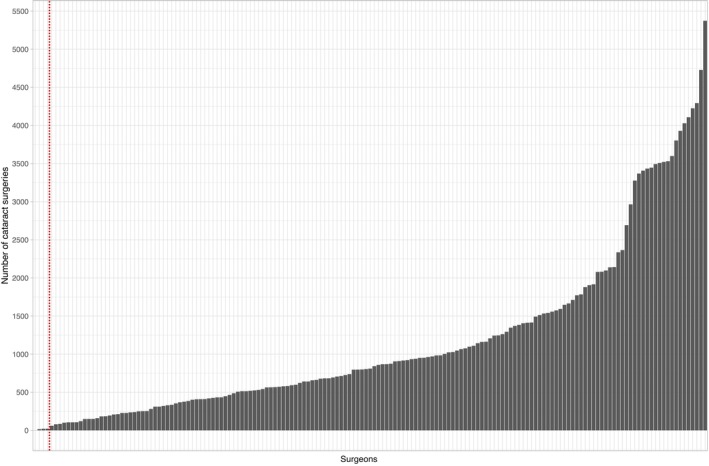
Surgical volumes. The histogram displays the distribution of surgical volumes among the participating surgeons for the study period, spanning 2021 to 2022, with a cut‐off at 10 annual surgeries.

The study included surgeons with varying levels of annual caseload, spanning from 11 to 2364 cases, median 421 cases per year. Figure 2 displays a histogram depicting the distribution of surgical volumes among the participating surgeons for the study period, spanning 2021 to 2022. The vast majority of surgeries (74.5%, *n* = 139 451) were performed by a surgeon with over 10 years of experience (Table 2). Surgeons who favoured the direct chop technique conducted a higher proportion of surgical teaching (35.0%) compared to those who favoured other techniques. The characteristics of surgeons and their respective cases are depicted in Table 2, with surgeons categorized according to their favoured surgical technique. The preferred techniques among less experienced surgeons, particularly those with 0–1 years of experience, were divide and conquer together with stop and chop, with these techniques being used in 100% of their cases (*n* = 1226).

**TABLE 2:** Surgical techniques for cataract extraction during 2021–2022, most favoured^a^.

Variable	Divide and conquer	Stop and chop	Direct chop	Tilt and tumble	All techniques
Number of surgeons, *n* (%)^a^	50 (27.0)	73 (39.5)	49 (10.3)	13 (7.0)	161/185^a^
Number of procedures, *n* (%)^a^, ^b^ Procedures/surgeon/year	44 607 (23.8)	84 016 (44.8)	73 138 (39.0)	18 717 (10.0)	187 182
Median (range)	326 (11–1902)	399 (50–2364)	549 (75–2147)	312 (53–1902)	421 (11–2364)
	***p* = 0.02** ^c^	*p* = 0.84^c^	***p* = 0.01** ^c^	*p* = 0.85^c^	
Gender of surgeon^a^	*n* = 50	*n* = 73	*n* = 49	*n* = 13	*n* = 161
Women, *n* (%)	19 (38.0)	26 (35.6)	8 (16.3)	4 (30.8)	49 (30.4)
	*p* = 0.20^d^	*p* = 0.23^d^	***p* = 0.01** ^d^	*p* = 1.00^d^	
Gender of cataract patients	*n* = 44 599	*n* = 83 974	*n* = 72 124	*n* = 18 714	*n* = 187 121
Women, *n* (%)	25 881 (58.0)	48 528 (57.8)	40 697 (56.4)	10 754 (57.5)	107 005 (57.2)
	***p* < 0.001** ^d^	***p* < 0.001** ^d^	***p* < 0.001** ^d^	*p* = 0.42^d^	
Age of patient at surgery, years	*n* = 44 599	*n* = 83 974	*n* = 72 124	*n* = 18 714	*n* = 187 121
Median (range)	75.0 (14–105)	76.0 (9–105)	76.0 (10–102)	75.0 (15–101)	76.0 (9–105)
	***p* = 0.02** ^c^	***p* < 0.001** ^c^	*p* = 0.40^c^	***p* < 0.001** ^c^	
Posterior capsular rupture	*n* = 44 599	*n* = 83 973	*n* = 72 122	*n* = 18 714	*n* = 187 118
Yes, *n* (%)	253 (0.6)	418 (0.5)	361 (0.5)	87 (0.5)	972 (0.5)
	*p* = 0.11^d^	*p* = 0.24^d^	*p* = 0.37^d^	*p* = 0.31^d^	
Surgical setting	*n* = 44 599	*n* = 83 974	*n* = 72 124	*n* = 18 714	*n* = 187 121
Public, *n* (%)	12 603 (28.3)	29 036 (34.6)	29 092 (40.3)	3498 (18.7)	67 974 (36.3)
Private, *n* (%)	31 996 (71.7)	49 791 (59.3)	37 682 (52.2)	15 110 (80.7)	108 544 (58.0)
Both public and private, *n* (%)	0 (0)	5147 (6.1)	5350 (7.4)	106 (0.6)	10 603 (5.7)
	***p* < 0.001** ^e^	***p* < 0.001** ^e^	***p* < 0.001** ^e^	***p* < 0.001** ^e^	
Type of cataract procedure	*n* = 44 599	*n* = 83 974	*n* = 72 124	*n* = 18 714	*n* = 187 121
Phacoemulsification with IOL in posterior chamber, *n* (%)	43 775 (98.2)	83 342 (99.2)	71 459 (99.1)	18 253 (97.5)	184 830 (98.8)
Other types of cataract procedures, *n* (%)	824 (1.8)	632 (0.8)	665 (0.9)	461 (2.5)	2291 (1.2)
	***p* < 0.001** ^d^	***p* < 0.001** ^d^	***p* < 0.001** ^d^	***p* < 0.001** ^d^	
Surgical tutoring	*n* = 44 599	*n* = 83 974	*n* = 72 124	*n* = 18 714	*n* = 187 121
Yes, *n* (%)	7101 (15.9)	17 593 (21.0)	25 279 (35.0)	874 (4.7)	48 340 (25.8)
	***p* < 0.001** ^d^	***p* < 0.001** ^d^	***p* < 0.001** ^d^	***p* < 0.001** ^d^	
Surgical experience	*n* = 44 599	*n* = 83 974	*n* = 72 124	*n* = 18 714	*n* = 187 121
0–1 year, *n* (%)	571 (1.3)	655 (0.8)	0 (0)	0 (0)	1226 (0.7)
2–5 years, *n* (%)	1604 (3.6)	6960 (8.3)	5709 (7.9)	4152 (22.2)	17 341 (9.3)
6–10 years, *n* (%)	6660 (14.9)	17 199 (20.5)	6273 (8.7)	679 (3.6)	29 103 (15.6)
>10 years, *n* (%)	35 764 (80.2)	59 160 (70.5)	60 142 (83.4)	13 883 (74.2)	139 451 (74.5)
	***p* < 0.001** ^e^	***p* < 0.001** ^e^	***p* < 0.001** ^e^	***p* < 0.001** ^e^	
Staining of the anterior capsule	*n* = 44 599	*n* = 83 974	*n* = 72 124	*n* = 18 714	*n* = 187 121
Yes, *n* (%)	1300 (2.9)	2636 (3.1)	1930 (2.7)	230 (1.2)	5106 (2.7)
	***p* = 0.006** ^d^	***p* < 0.001** ^d^	*p* = 0.27^d^	***p* < 0.001** ^d^	
Use of capsular retention hooks	*n* = 44 599	*n* = 83 974	*n* = 72 124	*n* = 18 714	*n* = 187 121
Yes, *n* (%)	308 (0.7)	416 (0.5)	347 (0.5)	25 (0.1)	985 (0.5)
	***p* < 0.001** ^d^	*p* = 0.09^d^	***p* = 0.03** ^d^	***p* < 0.001** ^d^	
Preoperative visual acuity	*n* = 44 557	*n* = 83 900	*n* = 72 068	*n* = 18 690	*n* = 186 964
Median (range), decimal	0.50 (0.01–1.95)	0.50 (0.01–1.95)	0.50 (0.01–1.95)	0.50 (0.01–2.00)	0.50 (0.01–2.00)
	*p* = 0.85^c^	*p* = 0.25^c^	***p* < 0.001** ^c^	***p* = 0.02** ^c^	

*Note*: Only surgeons with ≥10 procedures/year were included. Surgeons without any of the above‐mentioned techniques were excluded. Patients <8 years old or with unknown gender were excluded, procedures with missing surgical id were excluded (*n*=2). Bold indicated significance *p* < 0.05.

^a^ Some surgeons had more than one favourite method.

^b^ All procedures during 2021–2022.

^c^ Mann–Whitney *U*‐test.

^d^ Fisher's exact test.

^e^ Pearson Chi‐squared.

In terms of overall prevalence, the most frequently preferred method for cataract surgeries was stop and chop; the surgeons stating this as their preferred method accounted for 84 016 cases (44.9%). This technique was also the top choice among most surgeons, with 73 surgeons (39.5%) selecting it as their preferred method (Table 2). Surgeons who favoured the direct chop method were predominantly males (83.7%), exhibited a significantly higher tendency towards mentoring other surgeons (35.0%), and possessed substantial experience, with 83.4% having more than 10 years of surgical practice. Cases performed by surgeons who preferred the tilt and tumble approach were primarily conducted in a private healthcare setting (80.7%) and showed a distinctly lower use of capsular staining (1.2%) and capsular hooks (0.1%).

The yearly average procedure count showed the highest numbers in the direct chop group (with a mean of 736 and a median of 549, *p* = 0.01) and the tilt and tumble group (with a mean of 720 and a median of 312, *p* = 0.85). Notably, there was a significant difference between the mean and median in the tilt and tumble group, indicating a non‐normal distribution.

Our data did not indicate a noteworthy relationship between the occurrence of capsular rupture and the preference for a particular technique. The rate of capsular rupture was consistent at 0.5% (*n* = 972) across all groups, except in the divide and conquer group, where it was slightly higher at 0.6% (*p* = 0.11), although this difference was not statistically significant. This difference in rate of capsular rupture between different preferred surgical techniques remained non‐significant even when adjusting for possible confounding variables in a logistic regression model (Table 3). However, we observed a trend suggesting that the tilt and tumble technique might reduce the risk of capsular rupture, adjusted odds ratio 0.81 (95% CI, 0.65–1.02, *p* = 0.07). Low surgical volume (<400 cases/year), surgical setting (public only), preoperative visual acuity ≤0.1 (decimal), capsular staining and use of capsular hooks were significantly associated with incidence of capsular rupture.

**TABLE 3:** Multivariate analysis of predictors for posterior capsular rupture, including preferred cataract surgery technique.

Covariates	*B*	SE	OR	95% CI	*p*‐value^a^
Age, years	0.008	0.004	1.008	1.001–1.015	0.035
Gender, male	0.007	0.066	1.007	0.885–1.145	0.918
Preferred cataract surgery technique					
Divide and conquer	−0.105	0.076	0.901	0.776–1.046	0.170
Stop and chop	0.084	0.067	1.088	0.955–1.240	0.206
Direct chop	−0.002	0.071	0.998	0.868–1.147	0.976
Tilt and tumble	−0.209	0.115	0.811	0.647–1.016	0.069
Surgical volume, <400 procedures per year	0.553	0.078	1.738	1.492–2.025	<0.001
Experience, less than 2 years	0.466	0.229	1.594	1.017–2.498	0.042
Surgical setting, public only	0.882	0.081	2.416	2.060–2.833	<0.001
Preoperative BCVA, ≤0.1 (decimal)	0.861	0.087	2.367	1.995–2.807	<0.001
Capsular staining	0.911	0.105	2.488	2.023–3.059	<0.001
Use of capsular hooks	2.155	0.124	8.630	6.772–10.996	<0.001

Abbreviations: *B*, regression coefficient; BCVA, best corrected visual acuity; CI, confidence interval; OR, odds ratio; SE, standard error.

^a^ Binary logistic regression with posterior capsular rupture as dependent variable. A *p*‐value of <0.05 was considered statistically significant.

## 4 DISCUSSION

The primary objective of this study was to investigate the preferred phacoemulsification techniques among Swedish cataract surgeons and to analyse possible associations between surgical technique and level of experience, surgical volume and outcome, especially with regard to the rate of PCR.

Determining the most beneficial surgical technique for the patient is challenging because the Swedish National Cataract Registry does not document the specific technique used, and the cohort displays a wide range of surgical volumes (Figure 2). Furthermore, surgeons may employ various techniques and even combine them. By providing surgeons with different choices of techniques in our survey and asking them to specify the extent to which they use each technique as a percentage, we believe that we have successfully captured a comprehensive overview of how and to what extent different techniques are utilized in Sweden. Additionally, by linking the surgeons' IDs with the national patient data registry, we were able to gain valuable insights into the outcomes of patients operated on by surgeons with a preference for a particular technique.

The analysis of non‐responders indicated that the majority of cases from 2021 to 2022 were managed by the participating surgeons, accounting for 67.5%. Despite some slight discrepancies between the different groups, we have confidence that the responding surgeons effectively represent the entire cohort. Given the substantial size of the groups under scrutiny, 285 033 cases in total, even minor distinctions between the groups could potentially hold statistical significance.

Surgeons who favoured the divide and conquer and stop and chop techniques were more likely to be less experienced, with 0–1 years of surgical practice. These data suggest that the divide and conquer method is employed for training new surgeons and used in the early stages of their surgical careers. What was somewhat surprising was the extensive use of this method in private clinics, where surgeons typically have extensive experience. Evidently, many practitioners continue to rely on this technique throughout their careers. This indication is also supported by other survey studies (Bethke, 2022; ESCRS, 2021). Additionally, surgeons who preferred the divide and conquer approach were more likely to use capsular hooks, with a notable 0.7% rate, and commonly conducted surgeries in private clinics (71.7%).

Several other studies have previously examined the risk of PCR using the Swedish National Cataract Registry (Bro et al., 2024; Hård & Segerstad, 2022; Zetterberg et al., 2020). One of the key findings of our study was the lack of a significant association between the choice of surgical technique and the occurrence of PCR (posterior capsular rupture). The rate of PCR was consistent across all groups, except for a slightly higher rate in the divide and conquer group, which was not statistically significant. This finding suggests that while surgeons may have varying preferences for surgical techniques, these preferences do not appear to significantly impact the risk of PCR (Tables 2 and 3).

The trend suggesting that the tilt and tumble technique might reduce the risk of capsular rupture and the fact that surgeons favouring this technique had a lower tendency to use capsular hooks and staining of the capsule less frequently, may be indicative of a more gentle and controlled approach to cataract surgery, which could contribute to a reduced risk of capsular rupture. However, it is important to consider that since capsule staining is a sign of dense cataracts and the presence of hooks at the rhexis edge indicates loose zonules, both of these factors can be viewed as indicators of the complexity of cases (Blecher & Kirk, 2008; Jacobs et al., 2006; Venkateswaran & Henderson, 2022). Hence, it is possible that surgeons using the tilt and tumble technique had less complex cases. Nonetheless, it is important to acknowledge that the group of surgeons favouring tilt and tumble was relatively small (comprising 13 surgeons), and some of them had lower caseloads and also performed other types of surgeries (combined procedures). The lower caseload could contribute to a higher rate of capsular rupture (Bell et al., 2007; Zetterberg et al., 2020).

In addition to displaying the pattern of different surgical techniques among Swedish cataract surgeons, this study provides insights into the demographics and professional practices of Swedish cataract surgeons. Notably, 75% of the cataract surgeries in Sweden were performed by surgeons with more than 10 years of experience, indicating a high volume of cases performed by experienced surgeons. However, female surgeons remained a minority (30.9%), with their representation closely mirroring the number of cases performed by female surgeons in the country (25.6%). The proportion of women among all ophthalmologists in Sweden was 48.7% (711 of 1461 in total) in July 2023 (personal communication with the Swedish National Board of Health and Welfare). Additionally, a quarter of the surveyed surgeons were engaged in tutoring other surgeons during the period of 2021–2022.

There are no studies that comprehensively document the surgical techniques and habits of cataract surgeons. It appears that there is a gap in the knowledge in this area, which our study partially addresses. Previous survey studies with a low number of participants or significant uncertainty regarding the dissemination of information about the study exist (Bethke, 2022; ESCRS, 2021), but no studies linking a substantial amount of patient data from a registry are available.

Nevertheless, the available research yields similar results as in the present study regarding the utilization of the most common surgical techniques in phacoemulsification (Table 1). Chopping is by far the most common, followed by divide and conquer while tilt and tumble (supracapsular phaco) is used to a very limited extent (Bethke, 2022; ESCRS, 2021). The femtosecond laser technique is used to some extent in certain parts of the world, but in Sweden the technology is not used at all, which is why it was not included as a response option in the survey.

## 5 LIMITATIONS

It is important to acknowledge some limitations of the present study. First, this data were collected through a survey, which may be subject to self‐report bias. Second, this study was conducted over a limited time frame (2021–2022) and may not capture long‐term trends or evolving surgical practices. Finally, while various confounding factors were adjusted for, there may still be unmeasured variables that influence surgical outcomes.

We assessed the frequency of capsular rupture as a complication. Other complication parameters such as cystoid macular oedema, retinal detachment and secondary cataract were not assessed as these parameters are not available in the registry. Data on the incidence of endophthalmitis are available, but there are so few cases per year (2–4 cases per 10 000) that we considered an assessment would not be meaningful for this study.

The surgeons who participated in the study performed 67.5% of the cases during the specified time period. It may be considered a weakness that the study did not have a larger number of participants. However, given the low and uncertain participation rates in similar survey studies, and since an analysis of the cases that were not operated on by the responding surgeons was included, we view this number as a strength of the study.

A smaller subset of surgeons (10%) favoured the use of direct chop as their technique of choice. This group comprised 84% males and performed 40% of the surgeries. They also constituted the most experienced percentage (>10 years of experience) and operated on patients with the lowest visual acuity. These, as well as similar, types of confounders have been accounted for in our logistic regression analysis.

The survey responses provide insights into the individual preferences of surgeons regarding the utilization of a particular surgical approach. However, it remains uncertain whether the surgeons have definitively employed this approach in individual cases. Therefore, the precise technique applied in each specific case remains undetermined, only revealing the preferences of the surgeon performing the case. Additionally, it is plausible for a surgeon to express favour towards multiple techniques simultaneously and also, some surgeons might not even know the proper name for the technique they are using. If it had been possible with access to a registry that records the specific technique used in each individual procedure, maybe it would have contributed to further and more secure insights. In such a scenario, it would be possible to start by analysing the actual technique used in each case and, based on that, determine the surgeons' preference profiles and associated complication frequencies.

In summary, with this study, we have mapped the use of various surgical techniques in cataract surgery in Sweden, which appears to align with other survey studies. Furthermore, we have succeeded in demonstrating that the choice of surgical method does not appear to contribute to either an increased or decreased PCR rate.

## FUNDING INFORMATION

Fundings have been received from ‘Alerisfonden’ for Johan Ursberg.

## Supporting information


Data S1:
