# Supplementary material for: Phacoemulsification practices: A comprehensive analysis of the surgical landscape in Sweden 2021–2022
Source: Acta Ophthalmol. 2024 Aug 20;103(1):69–76. doi: 10.1111/aos.16754 (PMC11704840; doi:10.1111/aos.16754)
Supplement: Supplementary file 1 — Data S1: [file AOS-103-69-s001.docx]

**Survey – Surgical techniques**

This survey aims to map the surgical methods of Swedish cataract surgeons. In addition to your responses below, we will also use the cataract registry for further analysis.

The study has been approved by the ethics review board. The results will be presented at the group level. We are grateful for your participation!

A. How many years have you independently performed cataract surgery?

1. 0-1
2. 2-5
3. 6-10
4. > 10

B. Which techniques do you use? Indicate the extent to which you have used each technique during 2021 and 2022.

Divide and Conquer _________%

Stop and chop _________%

Direct chop _________%

Tilt and tumble/supracapsular faco _________%

Other technique …………………….. _________%

C. Have you mentored any new cataract surgeons in 2021 and 2022? Yes/No

I consent to participate in the study. (link to or attached participant information)

Name:

Clinic/Clinics:
